# Effectiveness of paranasal air suction on acute migraine using portable air sucker – a randomized, double blind study

**DOI:** 10.1186/s12883-021-02203-x

**Published:** 2021-04-24

**Authors:** S. M. R. Bandara, S. Samita, A. M. Kiridana, H. M. M. T. B. Herath

**Affiliations:** 1 Neurology Unit, Teaching Hospital Kandy, Kandy, Sri Lanka; 2 University of Peradeniya, Peradeniya, Sri Lanka; 3 Teaching Hospital Kandy, Kandy, Sri Lanka; 4 National Hospital of Sri Lanka, Colombo, Sri Lanka

**Keywords:** Migraine, Headache, Adolescents, Para nasal sinus air suction, Portable air sucker

## Abstract

**Background:**

Migraine is a primary headache disorder and is the most common disabling primary headache disorder that occurs in children and adolescents. A recent study showed that paranasal air suction can provide relief to migraine headache. However, in order to get the maximum benefit out of it, an easy to use effective air sucker should be available. Aiming to fulfil the above requirement, a randomized, double blind control clinical trial was conducted to investigate the efficacy of a recently developed low–pressure portable air sucker.

**Methods:**

Eighty-six Sri Lankan school children of age 16–19 years with migraine were enrolled for the study. They were randomly allocated into two groups, and one group was subjected to six intermittent ten-second paranasal air suctions using the portable air sucker for 120 s. The other group was subjected to placebo air suction (no paranasal air suction). The effect of suction using portable air sucker was the primary objective but side of headache, type of headache, and gender were also studied as source variables. The primary response studied was severity of headache. In addition, left and right supraorbital tenderness, photophobia, phonophobia, numbness over the face and scalp, nausea and generalized tiredness/weakness of the body were studied. The measurements on all those variables were made before and after suction, and the statistical analysis was performed based on before and after differences. As a follow–up, patients were monitored for 24-h period.

**Results:**

There was a significant reduction in the severity of headache pain (OR = 25.98, *P* < 0.0001), which was the primary outcome variable, and other migraine symptoms studied, tenderness (left) (OR = 289.69, *P* < 0.0001), tenderness (right) (OR > 267.17, *P* < 0.0001), photophobia (OR = 2115.6, *P* < 0.0001), phonophobia (OR > 12.62, *P* < 0.0001) nausea (OR > 515.59, *P* < 0.0001) and weakness (OR = 549.06, *P* < 0.0001) except for numbness (OR = 0.747, *P* = 0.67) in the treatment group compared to the control group 2 min after the suction. These symptoms did not recur within 24-h period and there were no significant side effects recorded during the 24-h observation period.

**Conclusion:**

This pilot study showed that low–pressure portable air sucker is effective in paranasal air suction, and suction for 120 s using the sucker can provide an immediate relief which can last for more than 24-h period without any side effects.

**Trail registration:**

Clinical Trial Government Identification Number – 1548/2016.

Ethical Clearance Granted Institute – Medical Research Institute, Colombo, Sri Lanka (No 38/2016).

Sri Lanka Clinical Trial Registration No: SLCTR/2017/018.

Date of registration = 29/ 06/2017.

Approval Granting Organization to use the device in the clinical trial– National Medicines Regulatory Authority Sri Lanka (16 Jan 2018), The device won award at Geneva international inventers exhibition in 2016 and President award in 2018 in Sri Lanka. It is a patented device in Sri Lanka and patent number was SLKP/1/18295.

All methods were carried out in accordance with CONSORT 2010 guidelines.

## Introduction

Migraine is a primary headache disorder and is the most common disabling primary headache disorder that occurs in children and adolescents. Recurrent headaches can badly impact school age children in several ways, including school stigma, school absences, deterioration of academic performance, and impaired ability to establish and maintain peer relationships [1] . Among headache disorders, migraine is known to be the most common cause of in children The prevalence of migraine headache in the USA ranged from 8 to 23% in adolescents [2]. Most of the oral medications for acute migraine usually take more than 30 min to become effective [3].Therefore, it will be useful to investigate a convenient treatment modality to reduce the severity of headache within a short period of time considering the pathophysiology of migraine. The previous study showed that sixty–second paranasal air suction could provide an immediate pain relief in acute migraine. The hypothesis in this method was that the suction of air from the paranasal sinuses removes or reduces the synthesis of the neuro and vasoactive air molecules that could be the causative agents for migraine [4]. However, in that study the sucker used was a standard sucker used in the hospital and it is not easy to carry and cannot be used by the patient himself or herself. Thus, if an easy to use sucker can be made available, all migraine patients can best benefit the pain relief from paranasal sucking. Moreover, in the previous work, we studied only the immediate effect, and effects and side effects within 24 h after suction were not studied [4]. Accordingly, objective of the current study was to investigate the effectiveness of the recently developed low–pressure portable air sucker on paranasal air suction and providing relief to migraine patients and observe the effects and side effects for 24 h.

## Methodology

**Fig. 1:**
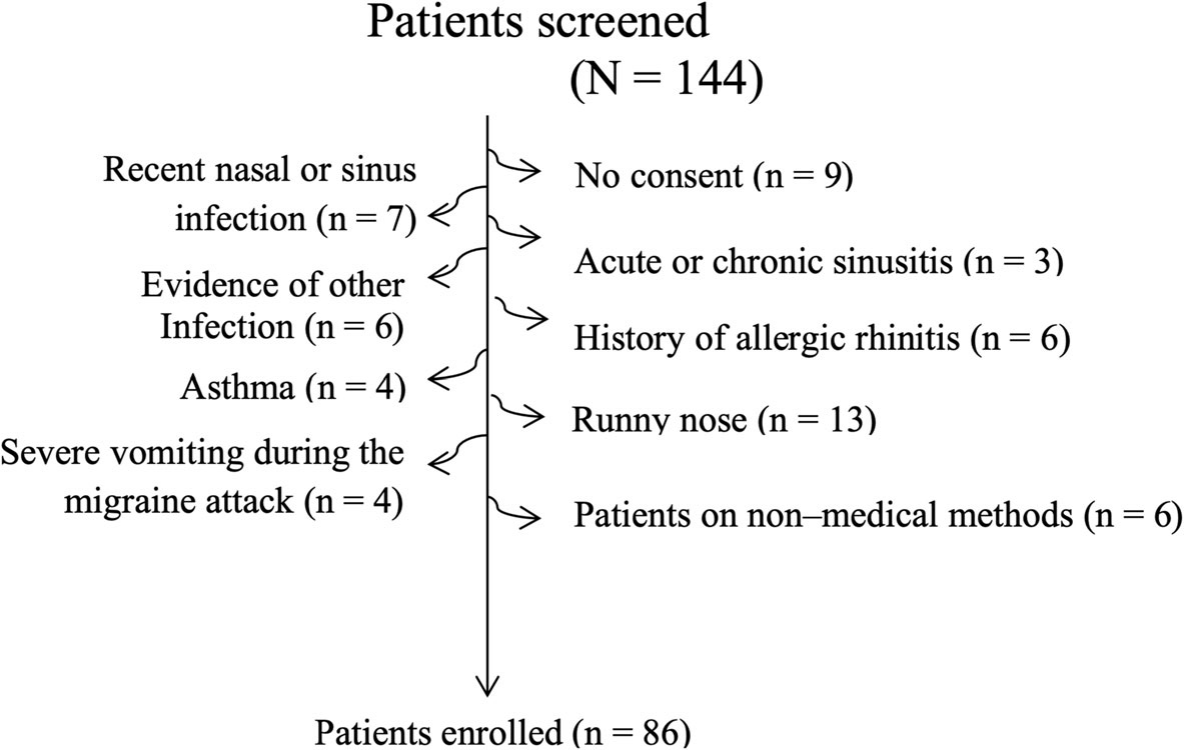
The patients enrollment flow chart

This randomized, parallel–arm, double–blind, prospective study adheres to CONSORT guidelines. All methods were carried out in accordance with CONSORT 2010 guidelines. The subjects were selected from two-stage randomization process (stage 1 being selection of schools randomly from Kandy district (an administrative unit) in Sri Lanka and stage 2 being selection of subjects randomly within the selected schools). A formal informed written consent was taken from all participants who were 18 years of age or above and from legally acceptable representative/guardian/parents of the participants who were below 18 years. The inclusion criteria were similar as the previous study [4] and included patients in the age group of 16–19 years, diagnosed with migraine according to the International Headache Society (IHS) criteria [5]. Patients with more than 3 migraine attacks but not more than 15 attacks per month, who have not taken an acute treatment during the testing period, were selected. Exclusion criteria were; history of intracranial lesion or tumor, recent nasal or sinus infection, acute or chronic sinusitis, evidence of another infection (i.e., acute otitis media or pneumonia), history of allergic rhinitis, asthma or an underlying immune deficiency, cystic fibrosis, immotile cilia syndrome, recent head and facial trauma, runny nose, severe vomiting during the migraine attack, smoking, alcohol or drug abuse, participants who were on hormonal therapy for any condition or illness, patients with psychiatric illness, patients on non–medical/non–nutritional treatment for migraine prevention such as acupuncture or psychotherapy, patients on fasting and had exercise or used any nasal drops or steam inhalation 1 h before the procedure. This flow chart was described in the Fig. 1. Patients who did not consent were also excluded. Since the key response variable was severity of headache, sample size was determined based on this variable. In order to detect headache pain drop difference of 30% between the two groups with the power of the test of 0.8 and type–I error rate of 0.05, required sample size was computed to be 38 for each group [6]. To minimize the bias due to dropouts, 43 patients were recruited for each group. The study was carried out from 07 March 2018 to 13 June 2018.

**Fig. 2:**
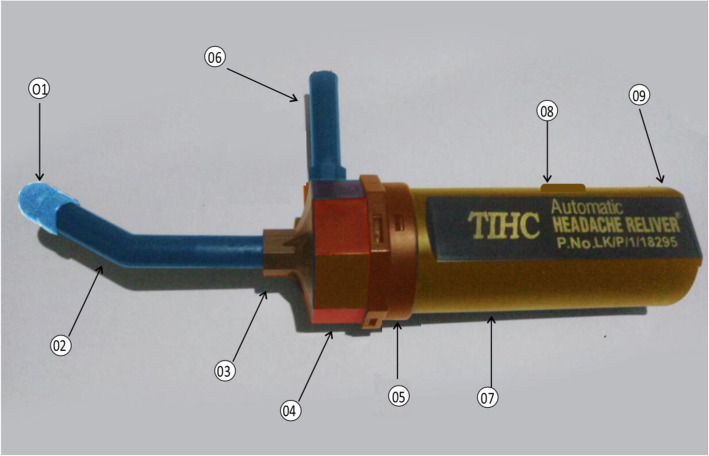
The portable sucker device. 01- Nasal cap – to fix in to the nasal cavity smoothly. 02 - Nasal suction arm – to suck out nasal and para nasal air out from the cavies in to the suction cavity part of the device. 03 - Inlet - fix nasal suction arm to the suction cavity part of the device. 04 - Head of the device - covers the rotator blades of motor part of the suction motor. 05 - Neck of the device – covers the motor of the device. 06 - Out let – Put out the sucked air. 07 - Cylinder or body part of the sucker – The housing of cylinder covers and hold the battery and the on and off switch of device. 08 - Switch of the device – To on and off the device when necessary. 09 - Closing lid - To close the end of cylinder with fixing of battery to the device

The selected children were examined at their own school premises. When the selected participants presented with typical migraine headache for more than 1 h, they were randomized into treatment (paranasal air suction) or control (placebo suction) group. All subjects were studied only once. The air suction devise tested in this study was a low – pressure portable air sucker (Fig. 2 with 12–15 mmHg suction capacity. The device is battery operated (four 1.5 V size AA). It is easy to carry and can be used by patient himself/herself.

The suction process was carried out similar to our previous research methodology [4]. During this process, nasal and paranasal sinus air were sucked six consecutive times from each nostril. Each suction was for 10–second duration with a 10 s suction free period between two suctions. Therefore, each subject was subjected to 120–second suction altogether. Participants were instructed to hold the breath by closing both nostrils by their own hand and then open one nostril for the suction for 10 s. They were allowed for mouth breathing if they need during the suction period. After 10 s of suction free period, they closed the opened nostril and opened the other nostril for air suction for 10 s. During suction, the suction tube did not contact the nostrils but we kept it in the air space of the outer part of nostrils, very close to nasal orifice. The control group was tested by keeping the same type of a nasal suction tube close to the nostrils. The appearance of the nasal air suction tube end was similar in both groups. They were also given the same instructions and were asked to close and open the nostrils in a similar manner. However, they were not exposed to air suction procedure but they were made to hear the sound of the air sucker. All these measures were taken to provide similar perception to both groups to reduce psychological bias.

The key symptom or the primary response variable studied was severity of headache (pain). In addition, left and right air flow rate, left and right scalp and supraorbital tenderness, photophobia, phonophobia, numbness over the face and scalp, nausea and generalized tiredness/weakness of the body were also studied as response variables. The primary endpoint was the headache pain relief. All response variables, except the numbness, were measured in ordinal scale and the examiners were also blinded. Air flow rates were measured on a scale of 0 to 3 based on the way the assessor felt the flow rate (0 = no flow, 1 = low flow, 2 = moderate flow and 3 = normal). The severity of the headache was measured using a standard pain rating scale/ Visual Analog Scale (0 being pain free and 10 being very severe pain) before and after the air suction procedure. Supraorbital and scalp tenderness was assessed by the same examiner applying pressure over the area until some blanching of their fingernail was discernible [7]. This was assessed on both right and left. The supraorbital notch (lying between the nasion and the trochlea) where the supraorbital branch of the ophthalmic nerve and the supratrochlear branch of the ophthalmic nerve were pressed to elicit supraorbital pain [7].The severity of tenderness felt to the subjects was measured using the above pain rating scale Photophobia, phonophobia, nausea and generalized tiredness/weakness were measured on a scale of 0 to 3 (0 = no symptoms, 1 = mild symptoms, 2 = moderate symptoms and 3 = severe symptoms). In order to measure photophobia, we exposed all participants to see the same bright light (using a same screen and illuminated for 30 s) that could be tolerated by healthy people (20 healthy subjects were exposed to different light intensities using the same screen and the lowest intensity they could tolerate for 30 s was taken). Phonophobia was measured by exposing all participants to listen to a high pitch noise using a headphone for 30 s that could be tolerated by healthy people (20 healthy subjects were exposed to different high pitch noise amplitudes using the headphone and the lowest amplitude they could tolerate for 30 s was taken). Numbness was measured based on presence or absence of numbness (subjective view). All endpoints, except for air flow rates, were assessed before and 2 min after application of suction using the device and was monitored at fixed intervals of 2, 6 and 24 h after air suction. The explanatory variables (factors) considered in the study were, gender, type of migraine (with aura or without aura), side of the headache (left, right, or bilateral), and group (sucked or not).

The side effects of air suction were also inquired at fixed intervals of 2, 6 and 24 h after air suction. For this, each participant was given a questionnaire containing a list of side effects and check boxes and was asked to check if a side effect was present. The side effects included sneezing, intolerance to suction and sound of the device, bleeding, nasal irritation, palpitation /increase in heartbeat, fear, and space was left for any other side effect. Blood pressures and heart rate were also measured before and after the procedure.

Descriptive statistical analysis outcome was used to describe the characteristics of the study population. Statistical analysis was carried out by fitting models. Specifically, since the response variables were in ordinal scale and several effects were studied simultaneously, analysis was carried out by fitting cumulative logit models [8, 9]. The effect of each factor after adjusting for all other factors was examined using the likelihood ratio type3 (LR Type3). The differences between levels of factors were studied using maximum likelihood estimates of the fitted models. In the study, all response variables were measured before and after the intervention on each patient. Thus, the difference between score before the intervention and 2 min after the intervention was used for the analysis. Since the pain and tenderness was measured using a scale of 0 to 10, the difference between score before and after the intervention were grouped into ordinal categories (≤2.5, >2.5 to ≤ 5.0, >5.0 to ≤ 7.5 and >7.5 to ≤ 10 ). Since the response variables photophobia, phonophobia, nausea and generalized tiredness/ weakness were measured on a scale of 0 to 3, each value of the difference between scores before and 2 min after the intervention were taken as a separate ordinal category. The variable numbness was measured as present or absent (two categories) and thus the variable was modeled by fitting ordinary logit models. When there was no interaction between factors and effects of only one factor was found, the difference between levels of the factor was further illustrated using non–parametric methods. In such a situation, especially when the factor had only two levels, the specific non–parametric method, Wilcoxon Rank Sum Test [10] was used. First, the score difference (before– after) for each patient was calculated and then using those score differences, two levels were compared by performing Wilcoxon Rank Sum Test.

## Results

We screened 144 patients with migraine symptoms for eligibility and out of them 86 satisfied the criteria for recruitment. All 86 were recruited in the study with possible dropouts but there were no dropouts Study period was 3 months. Out of 86 patients, 44 (51.2%) were males and 42 (48.8%) were females. In terms of type of headache, there were 14 (16.3%) patients with aura and 72 (83.7%) without aura. With respect to the side of headache, there were four categories as left, right, both left and right, and scalp. The distribution of subjects among the four categories were 17 (19.8%), 29 (33.7%), bilateral (44.2%) and 2 (2.3%). All response variables, except numbness, were ordinal, and summary measures (median and inter quartile range) of initial measurements (baseline values) are given in Table 1. With respect to the numbness, which was measured in nominal scale, occurrence of numbness is given in Table 1. Table 1 indicates that baseline values of outcome variables are almost exactly the same, and it is because the study subjects were divided in to two groups randomly.

**Table 1:** Descriptive statistics of baseline values of outcome variables

a) Ordinal scale measurements				
Variable	Treatment group		Control group	
	Median	IQR	Median	IQR
Air flow (left)	1	1	1	1
Air flow (right)	2	1	2	1
Tenderness (left)	8	0	8	2
Tenderness (right)	8	4	8	2
Pain	6	2	6	2
Photophobia	2	1	2	1
Phonophobia	1	1	1	1
Nausea	2	1	2	1
Weakness	1	1	1	1
b) Nominal scale measurements				
Variable	Occurrence			
	Treatment group		Control group	
Numbness	6/43 (11.95%)		5/43 (11.63)	

The Deviances and Type 3 Likelihood Ratio (LR) statistics from fitting models to 8 response variables are given in Tables 2 and 3. Small Deviance values relative to the degrees of freedom (df) and large associated probabilities indicate all fitted models are adequate. This adequacy implies that there are no two–way and other higher order interactions between those explanatory variables with respect to response variables considered in the study. Thus, there is no influence of type of headache, side of headache and gender on the effect of paranasal suction. According to LR Type3 statistics (Tables 2 and 3), with respect to all response variables, except numbness, group variable (treated or control) had a significant effect (*P* < 0.0001). Almost all patients did not have numbness and thus the effect of paranasal suction on numbness could not be demonstrated. Out of other explanatory variables, only gender had an effect (*P* = 0.031) but only on right side tenderness. Type of migraine and the side of the headache had no effect (*P* > 0.1) on any response variable. The Odds Ratio (OR) estimates and 95% confidence intervals for OR of the group variable (treatment vs control) with respect to all response variables are presented in Table 4.

**Table 2:** Deviance and Type 3 LR statistics for fitted models: Variables; tenderness (left side), tenderness (right side), pain and photophobia

Source	df	Tenderness (left side)		Tenderness (right side)		Pain		Photophobia	
		LR (Type3)	P	LR (Type3)	P	LR (Type3)	P	LR (Type3)	P
Type of migraine	1	1.53	0.2161	0.86	0.3534	0.86	0.3535	0.52	0.3114
Side of the headache	3	1.37	0.7125	0.65	0.885	1.31	0.7264	0.64	0.8464
Gender	1	0.05	0.8197	4.65	0.031	1.59	0.2068	0.42	0.2911
Group	1	74.36	<0.0001	78.18	<0.0001	19.11	<0.0001	100.59	<0.0001
Deviance		32.20	0.9988	38.84	0.9846	16.53	0.9991	39.62	0.9999
		(60df)		(60df)		(38df)		(82df)

**Table 3:** Deviance and Type3 LR statistics for fitted models: Variables; phonophobia, numbness, nausea/vomiting and generalized tiredness/weakness

Source	df	Phonophobia		Numbness		Nausea		Weakness	
		LR (Type3)	P	LR (Type3)	P	LR (Type3)	P	LR (Type3)	P
Type of migraine	1	0.83	0.3613	0.66	0.4153	0.17	0.6766	0.55	0.4599
Side of the headache	3	0.77	0.8561	4.92	0.1778	4.61	0.2028	2.15	0.5410
Gender	1	0.05	0.8260	0.13	0.7207	2.11	0.1463	0.27	0.6029
Group	1	29.47	<0.0001	0.18	0.6683	88.62	<0.0001	83.44	<0.0001
Deviance		54.54	0.6745	18.37	0.3029	51.34	0.9968	30.41	0.9995
		(60df)		(16df)		(82df)		(60df)

**Table 4:** Odds Ratios from the fitted models for the factor group with respect to response variables

Response variable	Parameter	OR	Wald 95% Confidence Limits	
Tenderness (left)	Control vs Treatment	289.69	32.580	2575.5
Tenderness (right)	Control vs Treatment	267.17	42.513	1679.1
Pain	Control vs Treatment	25.982	3.2078	210.42
Photophobia	Control vs Treatment	2115.6	122.68	36,487
Phonophobia	Control vs Treatment	12.619	4.5694	34.845
Numbness	Control vs Treatment	0.747	0.196	2.849
Nausea	Control vs Treatment	515.59	54.533	4874.6
Tiredness/weakness	Control vs Treatment	549.06	52.926	5695.3

All ORs in Table 4 are greater than unity with *P* < 0.0001 except for numbness and thus they suggest the cumulative probability for smaller difference (before – 2 min after procedure) is higher for the control group compared to the treated group. In other words, higher difference in scores has been observed in treated patients compared to the controls. Since the difference was taken as (before – after) and the OR being greater than unity indicate that there has been a decrease in the score after suction in the treatment group. Moreover, all OR estimates, except for numbness, being large (12.619) indicate that there has been a huge drop in all pain measurements studied after suction. This fact was further confirmed by the 95% CI for ORs with minimum lower limit being 3.208. Regarding the effect of gender on right side tenderness, the maximum likelihood estimates for male relative to female on the right tenderness was 1.242 with the standard error of 0.5982. The corresponding OR estimate is 3.462 and the 95% CI for the OR is 1.072 to 11.183. This indicates that the decrease of pain in right side tenderness was higher in females compared to that of males. However, since this gender effect was observed only for the right side tenderness, further research may be necessary to investigate the consistency of this gender effect.

Since there was no interaction between factors and only the effect of group was consistent, Wilcoxon Rank Sum Test was also performed to compare the two groups, the control group and the treated group with respect to all response variables (Tables 5). The mean score (average rank) for the difference (before suction– after suction) of the two groups, Wilcoxon Rank sum Teat Statistic (S) and significant level probabilities (P) for each response variable are presented in Table 5. According to Tables 5, median of the differences is significantly higher in the treated group than that in the control group (*P* < 0.0001) for all variables except numbness. This outcome is consistent with the outcome from fitting cumulative logit models and this confirms the fact that paranasal suction substantially decreases tenderness, pain, photophobia, phonophobia, nausea and tiredness/weakness.

**Table 5:** Summary of Wilcoxon Rank Sum Analysis for the control and treated groups with respect to variables left side tenderness, right side tenderness, pain and photophobia

Group	Mean score							
	Tenderness (left)	Tenderness (right)	Pain	Photophobia	Phonophobia	Numbness	Nausea/vomiting	Tiredness/ Weakness
Control	22.63	24.98	22.60	22.97	30.35	44	23.74	24.35
Treatment	64.37	62.02	64.39	64.03	56.65	43	63.26	62.65
S	973	1074	972	987.5	1305	1892	1021	1047
*P*	<0.0001	<0.0001	<0.0001	<0.0001	<0.0001	0.9999	<0.0001	<0.0001

S denotes the Wilcoxon rank sum test statistic and *P* denotes significant level probabilities

Out of participants, only one participant had sneezing during 24-h observation period after air suction. In fact, no other side effects listed in the methodology or any other unusual outcomes were observed with participants. None of the participants who got relief from the headache did not have recurrence of headache or any other symptoms of migraine during the 24-h period so the analysis were similar at 24 h.

## Discussion

The study showed that paranasal air suction gave significant relief to adolescents with acute migraine. The findings of this study were consistent with the previous study [3]. In this study, other than the severity of headache (pain), relevant other responses were also evaluated. Of the responses made, supraorbital tenderness in migraine patients must be due to hypersensitivity of the trochlea and supraorbital nerves [11]. Cutaneous allodynia due to sensitization of central pain pathways is common in migraine and results in scalp tenderness [12]. In majority of cases scalp allodynia occurred at the height of headache [13]. In our study there was a significant reduction in scalp and supraorbital tenderness in the treated group suggesting that this intervention might have reduced the mechanism of cutaneous allodynia. Even triptans, if given early (within 1 h of migraine) can abort cutaneous allodynia in migraine patients [14] and triptans acts by inhibiting the release of vasoactive peptides, promoting vasoconstriction and reducing the levels of calcitonin gene related peptide [15]. Paranasal air suction might also remove these vasoactive substances, like NO, involved in migraine pathology and reduce the cutaneous allodynia [4].

Most patients with migraine have photophobia and phonophobia [16]. Wolff postulated that irritation of the region supplied by the ophthalmic division of the trigeminal nerve could facilitate photophobia [17]. Another hypothesis is that attenuation of inhibitory influences involved in the perception of pain in migraine patients leads to general hyper excitability of the special senses causing photophobia and phonophobia [18]. In our study paranasal sinus suction reduced both photophobia and phonophobia. The pathology of nausea and vomiting in migraine is not well understood but it has been shown that activation of the nucleus tractus solitarius, dorsal motor nucleus of vagus, nucleus ambiguus and periaqueductal gray area is associated with nausea and vomiting in migraine patients [19]. Generalized weakness and tiredness, numbness of the face and scalp are common postdrome symptoms of migraine [20, 21]. Preventive medications that reduce the frequency and the intensity of the headache has also reduced these symptoms [19]. These symptoms were also greater in patients with typical migraine and when the headache intensity is high [20, 21]. So a similar pathology that leads to central sensitization in migraine might play a role in generating these symptoms [20, 21]. Many vasoactive neuropeptides such as substance P, neurokinin A [22] calcitonin gene–related peptide [23], NO [24] and serotonin [25] have been hypothesized in migraine pathology and these same molecules can play a role in photophobia, phonophobia, nausea, vomiting, numbness and generalized tiredness/weakness. In our study, para nasal suction significantly reduced these symptoms along with the intensity of the headache so we can assume that para nasal air suction might have removed or reduced synthesis these vasoactive substances involved in migraine pathology and associated symptoms.

During the post procedure 24–hour period none of the patients had recurrence of headache or associated symptoms. Since the procedure was done within 1 h of onset of migraine, we can assume that early removal of causative molecules by paranasal air suction might have stopped the progression of the pathology of migraine at the onset. Even with Triptans, when used early at the onset of migraine shows higher incidence of pain–free status and lower incidences of early headache recurrence [26, 27]. In fact, we assumed that suction helps to ventilate paranasal sinuses with 21% oxygen that in turn helps to inhibit nitric oxide synthase enzyme since excess NO is produced when the oxygen concentration in paranasal cavities is low [24]. In addition air suction may help to increase cilliary beating and relive obstruction in the sinus tract pathway [28]. Both these effects might help to reduce synthesis and stagnation of potent neuroactive and vasoactive molecules (like sNO) and to improve or inhibit hypoxia and excess NO induced inflammation in the intranasal mucosa, ostial tract and paranasal sinuses.

In this study we used a potable low–pressure sucker, which is easy to transport and use. The findings of this study being consistent with the previous study and thus it is clear that portable air sucker is as effective as the standard sucker used in the hospital. Another advantage was that other than sneezing in one patient, there was no side effects of this procedure within the 24–hour observation period. The fewer side effects were due to exclusion of a risk of infective, allergic and traumatic cases from the study. In addition a low – pressure portable air sucker with 12–15 mmHg suction capacity was used. The latter vacuum pressure is below the level of harmful range of the air section. In fact the suction fan of the device did not exceed 80–120 mmHg vacuum, which is within the physical safety limits even for tracheostomy suction [29]. However, it is advisable not to use this type of device on patients who have a history of nasal bleeding or a recent nasal surgery, ear surgery or trauma to the nasal and para nasal area. Most importantly, this is not to be used in any condition with pus or mucus discharge or runny nose because it is not designed to collect mucus or pus.

As for limitations in this study, even though we used a rating scale, all the response variables we evaluated were subjective. The same assessor measured pain and tenderness and this could lead to performance bias for the response variables assessed. Also the procedure we used in the study cannot directly remove para nasal air and we assumed that suction of air from the nasal cavity might remove air molecules in the para nasal sinus cavities by the syphon effect. Another limitation of the study is that even though this study was based on that paranasal air molecules are responsible for migraine pathophysiology, we could not get sample from the air sucked out. However another study is already started by the authors to evaluate the air samples that were sucked out from the para nasal sinuses. The migraine physiopathology is multifactorial and complex and local air molecules (such as NO) synthesis cannot explain all mechanisms, but the main message from this study is that paranasal air suction can provide relief for acute migraine and future studies are needed to understand the mechanism of this. In this study all the participants were from a single district in Sri Lanka in the age group of 16–19 years. However, pathophysiology of migraine might be common and will not vary with age and geographical areas but we suggest more studies involving larger sample size with patients from different geographical areas and in different ages to evaluate the beneficial effects of paranasal suction in migraine patients. We would also suggest future research for chronic migraine patients and also evaluate this intervention as a method of prophylaxis in migraine.

## Conclusion

This pilot study showed that low-pressure portable air sucker evaluated in the study has provided the expected relief to migraine patients, as in the previous study. This study further showed that the intervention not only reduced severity of headache, left and right scalp and supraorbital tenderness, but also reduced the severity of photophobia, phonophobia, and nausea and generalized tiredness/weakness of the body in migraine patients. Moreover, the beneficial effects of suction were consistent, regardless of type of headache, side of headache and gender. In addition, it was very important to find no recurrence of headache and other symptoms and no significant side effects within the 24–hour follow up period. Further studies with more heterogenic samples could be useful to further validate these results and to understand the mechanism.

## Data Availability

The datasets supporting the conclusions of this article are included within the article.

## Abbreviations

NO: Nitric oxide
LR Type3: Likelihood ratio type3
